# Phenotypic and genetic aspects of epithelial barrier function in asthmatic patients

**DOI:** 10.1016/j.jaci.2017.04.005

**Published:** 2017-06

**Authors:** Matthew Loxham, Donna E. Davies

**Affiliations:** Clinical and Experimental Sciences and the Southampton NIHR Respiratory Biomedical Research Unit, University of Southampton Faculty of Medicine, Sir Henry Wellcome Laboratories, University Hospital Southampton, Southampton, United Kingdom

**Keywords:** Asthma, tight junction, innate immunity, cytokine, homeostasis, AhR, Aryl hydrocarbon receptor, AJ, Adherens junction, BHR, Bronchial hyperresponsiveness, CDHR3, Cadherin-related family member 3, DC, Dendritic cell, DUOX1, Dual oxidase 1, EGFR, Epidermal growth factor receptor, eQTL, Expression quantitative trait locus, GC, Gene cluster, GST, Glutathione-S-transferase, GWAS, Genome-wide association study, ILC, Innate lymphoid cell, ILC2, Type 2 innate lymphoid cell, NK, Natural killer, ORMDL3, Orosomucoid-like 3, PCDH1, Protocadherin 1, SC, Subject cluster, SNP, Single nucleotide polymorphism, TJ, Tight junction, TLR, Toll-like receptor, TSLP, Thymic stromal lymphopoietin

## Abstract

The bronchial epithelium is continuously exposed to a multitude of noxious challenges in inhaled air. Cellular contact with most damaging agents is reduced by the action of the mucociliary apparatus and by formation of a physical barrier that controls passage of ions and macromolecules. In conjunction with these defensive barrier functions, immunomodulatory cross-talk between the bronchial epithelium and tissue-resident immune cells controls the tissue microenvironment and barrier homeostasis. This is achieved by expression of an array of sensors that detect a wide variety of viral, bacterial, and nonmicrobial (toxins and irritants) agents, resulting in production of many different soluble and cell-surface molecules that signal to cells of the immune system. The ability of the bronchial epithelium to control the balance of inhibitory and activating signals is essential for orchestrating appropriate inflammatory and immune responses and for temporally modulating these responses to limit tissue injury and control the resolution of inflammation during tissue repair. In asthmatic patients abnormalities in many aspects of epithelial barrier function have been identified. We postulate that such abnormalities play a causal role in immune dysregulation in the airways by translating gene-environment interactions that underpin disease pathogenesis and exacerbation.

**Glossary**

**CIGARETTE SMOKE**

Gases, hydrocarbon vapors, and particulate matter generated by burning tobacco. Cigarette smoke contains around 4000 substances, more than 60 of which have been identified as carcinogens. Cigarette smoke promotes increased local elastase production, which contributes to lung tissue injury likely caused by increased epithelial permeability through loss of tight junction integrity.

**DEFENSINS**

A distinct family of antimicrobial peptides produced by epithelial cells of mucosal surfaces, as well as by neutrophils, natural killer cells, and cytotoxic T lymphocytes. Defensins have direct antimicrobial activity, as well as the ability to activate inflammatory responses.

**EPISTASIS**

The expression of one gene is influenced by the expression of 1 or more independently inherited (nonallelic) genes.

**GLUTATHIONE**

An intracellular antioxidant whose principal site of synthesis is the liver. Glutathione serves as a cofactor for glutathione peroxidase, which is responsible for detoxifying lipid peroxides.

**INNATE LYMPHOID CELLS**

Cells possessing lymphoid morphology but without antigen receptors. Their subpopulations are divided into groups that resemble T helper subsets. Group 2 innate lymphoid cells produce type 2 cytokines, such as IL-4, IL-5, IL-9, and IL-13, on stimulation with epithelium-derived cytokines, such as IL-33, IL-25, and TSLP.

**LATE-ONSET ASTHMA**

Often defined as asthma that develops in adolescence or adulthood. Most cases of asthma are diagnosed in childhood. Late-onset asthma tends to be more common in women than in men. Occupational asthma and aspirin-exacerbated respiratory disease are subtypes of late-onset asthma. Smoking and passive smoke exposure appear to be risk factors for late-onset asthma.

**NOD-LIKE RECEPTORS (NLRs)**

A family of cytoplasmic receptors that recognize bacterial products, such as bacterial peptidoglycan. NLRs include an N-terminal effector region and a central nucleotide oligomerization domain (NOD), and most have C-terminal leucine-rich repeats for ligand (PAMP) binding. Once activated, NODs assemble signaling proteins, resulting in nuclear factor κB and mitogen-activated protein kinase activation, and control the activation of inflammatory caspases.

**PARTICULATE MATTER (PM)**

PM is a term describing airborne dust particles originating from a range of sources and processes, such as fossil fuel combustion, waste incineration, cigarette smoking, and erosion, which can contain black carbon (soot), metals, polyaromatic hydrocarbons, and anions, such as sulfate and nitrate, among others. If sufficiently small to be inhaled (aerodynamic diameter <10 μm), PM can settle in the airways and exert a range of effects.

**PATHOGEN-ASSOCIATED MOLECULAR PATTERN (PAMP)**

Molecules associated with groups of pathogens that are recognized as “danger signals” by pattern recognition receptors, such as Toll-like receptors or NOD-like receptors. Many types of molecules can serve as PAMPs, including LPSs, peptidoglycans, lipoteichoic acid, nucleic acid variants, and flagellin.

**POLYAROMATIC HYDROCARBONS (PAHs)**

Also known as polycyclic aromatic hydrocarbons, PAHs are volatile substances produced by cooking oils and coal-burning or petroleum products. Indoor biomass burning generates PAHs and is associated with chronic obstructive pulmonary disease development in women.

**PSEUDOSTRATIFIED**

Although showing features of layering in an epithelium, all cells are still attached to the basement membrane.

**TOLL-LIKE RECEPTORS (TLRs)**

TLRs are a family of innate pattern recognition receptors that respond to a variety of structurally conserved molecules derived from pathogens (ie, PAMPs). They are single, membrane-spanning, noncatalytic receptors whose signaling pathways are finely regulated by Toll/IL-1 receptor homologous region (TIR) domain–containing adaptors. Differential use of these adaptor proteins provides specificity of individual TLR-mediated signaling pathways.

*The editors wish to acknowledge Daniel Searing, MD, for preparing this glossary.*

## Asthma heterogeneity

Asthma is a common chronic inflammatory disorder of the conducting airways, which undergo distinct structural and functional changes leading to nonspecific bronchial hyperresponsiveness (BHR) and variable airflow obstruction. Recruitment and careful clinical characterization of large cohorts of asthmatic patients has established beyond doubt that asthma is a heterogeneous disease in terms of phenotype, endotype (ie, underlying pathogenic mechanism), response to treatment, and/or long-term clinical outcomes.^1^ Cluster analysis has enabled identification of 4 to 5 phenotypic clusters that have differences in sex, asthma onset, lung function, atopic status, asthma control, health care use, and exacerbation frequency.^2^, ^3^, ^4^, ^5^ Molecular phenotyping of blood, induced sputum, and epithelial brushings has identified additional heterogeneity, especially in patients with severe asthma,^6^, ^7^, ^8^, ^9^ who are a major economic burden on the health care system because of poor responses to traditional asthma medications. Some of the differences in asthma clusters might reflect underlying genetic differences; for example, there are differences in genetic risk in early-onset compared with ***late-onset asthma***,^10^ whereas others might reflect differences in environment and lifestyle or, perhaps most likely, a combination of both gene and environment effects.^11^ Many, but not all, asthmatic patients have T_H_2 inflammation in their airways, and clinical trials with mAbs to IL-5, IL-13, or IL-4 receptor (α chain) have identified a type 2 endotype.^12^ Thus patient stratification with type 2 relevant biomarkers has enabled effective targeting of these treatments to subsets of patients with moderate and severe asthma.^13^, ^14^, ^15^, ^16^, ^17^ However, although clinical trials have shown that type 2 inflammation is an important disease modifier in some patients, they have also highlighted that non–type 2 inflammatory pathways must contribute to certain forms of asthma.^18^ These can include pathways associated with obesity or neutrophilia or with susceptibility to environmental factors, such as infection and air pollution, but disease mechanisms/endotypes are not well understood. We postulate that a dynamic interaction between a genetically susceptible epithelium and environmental risk factors for asthma is important for the development of asthma and its subphenotypes.^19^

## Bronchial epithelial barrier structure and function

Given the multitude of challenges imposed on the airway epithelium, it is not surprising that it combines structural and functional protective mechanisms with innate immunologic mechanisms to maintain healthy barrier homeostasis and to minimize inflammation and cellular dysregulation. Structurally, the bronchial epithelium is ***pseudostratified***, comprising mainly columnar multiciliated, secretory (goblet), and undifferentiated cells that overlie smaller basal cells with the capacity for self-renewal.^20^ Rare cell types include pulmonary neuroendocrine cells^21^, ^22^ and brush (tuft) cells^23^ that can have neurosensory or chemosensory functions, but information on these cells is limited.

On the epithelial surface, the mucociliary apparatus is a crucial primary innate defense mechanism that protects the lungs from deleterious effects of inhaled pollutants such as noxious gases and ***particulate matter,*** allergens, and pathogens. Surface epithelial cells and submucosal glands produce secretions comprising a superficial gel or mucus layer and a layer of periciliary fluid that contacts the epithelial surface. Mucus contains hydrated gel-forming mucins and a range of host defense and cytoprotective molecules, including ***defensins***, IgA, lactoperoxidase, catalase, superoxide dismutase, and low-molecular-weight antioxidants.^24^ The viscoelastic properties of the mucus are dictated in large part by the oligomeric secreted mucins MUC5AC and MUC5B,^25^ multifunctional glycoproteins that provide the structural framework of the mucus barrier. These bronchial secretions shield the epithelial surface, detoxify noxious agents, and trap many inhaled particles, allowing clearance through the action of the mucociliary escalator. MUC5B might also contribute to immune homeostasis by means of direct regulation of leukocyte functions.^26^, ^27^

**Fig 1:**
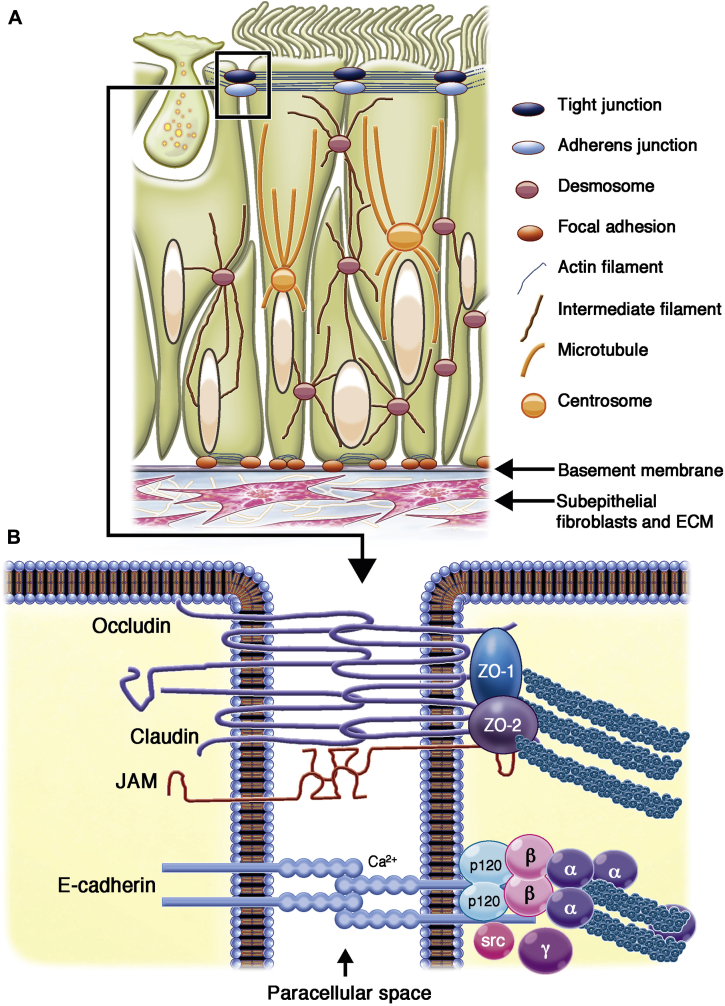
**A,** Schematic representation of a pseudostratified bronchial epithelial cell layer (comprising a goblet cell, 2 ciliated cells, and 2 basal cells) showing the junctional complexes and their interactions with the cytoskeleton or basement membrane to form a robust sheet-like structure. **B,** Illustration of the TJ and AJ complexes showing how they mediate cell-cell contact and interact with the actin cytoskeleton. *ECM*, Extracellular matrix; *JAM*, junctional adhesion molecule; *ZO*, zonula occludens.

In addition to secreting mucus, the bronchial epithelium forms a sheet-like structure that acts as a physical barrier to protect the internal milieu of the tissue. Individual epithelial cells contact each other through a range of cell-cell adhesion complexes (tight junctions [TJs], adherens junctions [AJs], and desmosomes) that control the permeability of the epithelial sheet and link with the cytoskeleton to resist mechanical stress (Fig 1); in addition, gap junctions directly connect the cytoplasm of adjacent cells, allowing cell-cell communication.^28^, ^29^, ^30^ The apical-most adhesive complexes are the TJs, which are formed by transmembrane and intracellular proteins that link to the actin cytoskeleton (Fig 1, *B*).^31^ TJs seal the epithelium, regulating paracellular passage of ions, water, and various macromolecules. They also maintain cell polarity by preventing lateral diffusion and intermixing of molecules in the apical membrane with those in the lateral membrane. Proteins of the TJs include tricellulin and occludin, which regulate the passage of macromolecules through the TJs,^32^ and claudins, which are responsible for the size- and charge-selective conductance properties of the TJ paracellular pathway.^33^

Expression of barrier or sealing claudins that selectively decrease paracellular cation permeability has been reported in normal human adult lung (claudins 1, 3, 4, 5, 7, and 18),^34^ and the expression profile varies with anatomic location and function.^35^, ^36^ Claudin-2, a pore-forming claudin, is also detected in the lung, and its presence is thought to increase ionic permeability by acting as a cation-selective pore.^36^

Located below the TJs are the AJs, which link to the actin cytoskeleton^37^, ^38^; desmosomes, which link to the intermediate filaments^39^; and hemidesmosomes,^40^ containing α_6_β_4_ integrins that facilitate attachment to the basement membrane (Fig 1, *A*). AJs and desmosomes are critical for providing the adhesive force to ensure the integrity of the cell layer. Cadherin-catenin complexes comprise the core of the AJs, bridging neighboring cells and the actin-myosin cytoskeleton and contributing to mechanical coupling between cells. In addition to its adhesive function, E-cadherin physically interacts with several receptor tyrosine kinases and affects their signaling abilities. Similarly, β-catenin, which is an integral structural component of the AJs, is also the key nuclear effector of canonical Wnt signaling in the nucleus.^41^ This coupling of cell-cell adhesion with signaling functions ensures that AJs can be extremely plastic, allowing the cell to adapt rapidly to its changing environment. Like AJs, the TJ plaque also contains many signaling molecules,^42^, ^43^ allowing proteins involved in cell-cell and cell-matrix adhesion to integrate and coordinate epithelial responses.^44^ Therefore perturbation in the turnover and concentration of junctional proteins is likely to have important implications for the maintenance and stability of the epithelium and the permeability barrier.

Junctional adhesion molecules also serve as sites for interaction of the epithelium with cells involved in immune surveillance. For example, TJ proteins interact directly with dendritic cells (DCs) to allow them to sample the airway lumen without disruption of the epithelial barrier,^45^, ^46^ whereas E-cadherin is a ligand for α_E_β_7_ integrin (CD103)–expressed T cells^47^, ^48^ and DCs.^49^ In addition to structural adhesion molecules, the bronchial epithelium expresses inducible adhesion molecules, such as intercellular adhesion molecules 1 and 2, which have essential functions in the clearance of T cells from the lung during resolution of inflammation.^50^

**Fig 2:**
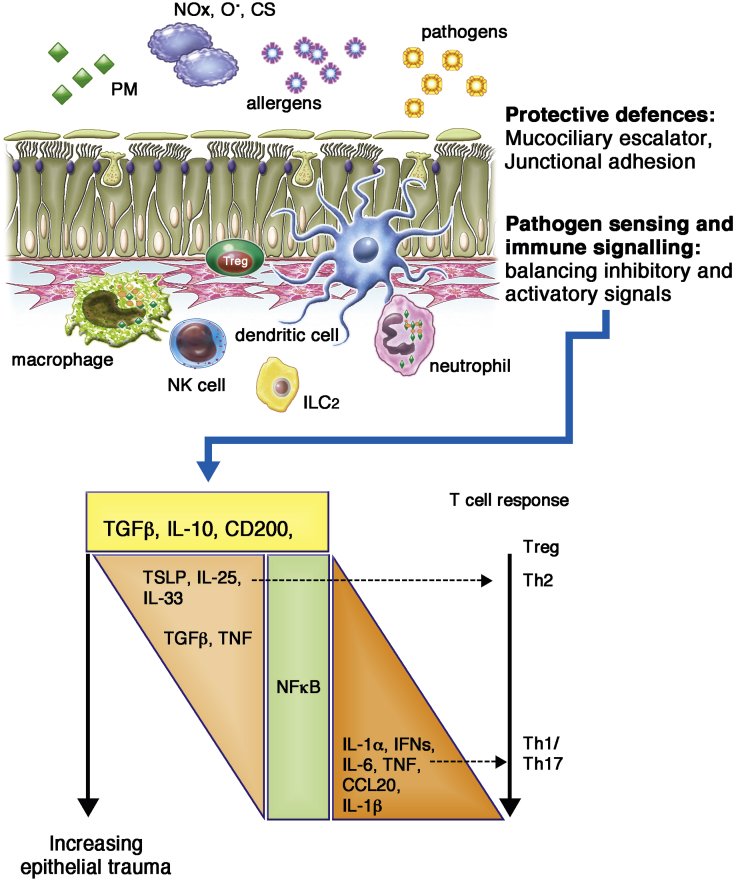
Schematic representation of epithelial barrier function illustrating protective and immunoregulatory functions. Under basal conditions, the epithelium maintains homeostasis by limiting exposure of the airway tissue to components of the inhaled environment and by balancing immunoregulatory signals. However, when compromised, the epithelium responds by releasing innate cytokines that help to orchestrate appropriate innate and adaptive immune responses. *CS*, Cigarette smoke; *NO*_*x*_, nitrogen oxides; *O*^*·*^, oxygen radicals; *PM*, particulate matter; *Treg*, regulatory T cell.

Airway epithelial cells express an array of pattern recognition receptors, including ***Toll-like receptors (TLRs)***, ***NOD-like receptors***, retinoic acid–inducible gene I (RIG-I)–like receptors, and a variety of natural killer (NK) cell receptor ligands. These enable detection of a wide variety of microbial and nonmicrobial agents, resulting in production of many different soluble and cell-surface molecules, collectively termed the epimmunome (cytokines, chemokines, damage-associated molecular pattern molecules, and major histocompatibility complex [MHC] gene products),^51^ that recruit and activate cells, such as macrophages and neutrophils, involved in inflammation and induction of adaptive immunity. Together, these responses enable many infections to be controlled by the immune system with limited damage to host tissues; however, it is important to note that both innate and adaptive immune-signaling events are involved in mediating tissue damage.^52^ For example, macrophages, neutrophils, and eosinophils release a range of molecules, including cytotoxic cytokines, cationic proteins, lipid mediators, metalloproteinases, and reactive oxygen species, that induce tissue damage or malfunction. Therefore the ability of the epithelium to control the balance of inhibitory and activating signals is essential not only for initiating an appropriate immune response to environmental challenges, if required (Fig 2), but also for temporally orchestrating these responses to limit tissue injury and control the resolution of inflammatory reactions through cell-surface molecules and release of inhibitory cytokines and lipids during tissue repair.

*In vitro* and *in vivo* studies have shown that epithelial cells can modulate a variety of immune cells. For example, epithelium-derived TGF-β is chemoactive for ***innate lymphoid cells*** (ILCs),^53^ which might provide early defense against pathogens and intervene in repair of damaged tissues. TGF-β secreted by bronchial epithelial cells has a direct inhibitory effect on T-lymphocyte proliferation, and epithelial cell–conditioned T lymphocytes show increased differentiation toward IL-10–producing T_R_1 cells.^54^ Epithelial cell secretions also inhibit proinflammatory responses of monocytes, macrophages, and DCs; increase DC expression of the negative regulatory programmed death ligand 1 (CD274); decrease the ability of DCs to induce T-lymphocyte proliferation^54^; and suppress human lung mast cell histamine secretion.^55^ Epithelial cells express CD200, which binds to the inhibitory immune receptor CD200R, which is expressed at high levels on lung macrophages. This not only maintains a strong threshold for response in the context of inhaled nonpathogenic antigens^56^ but also dampens macrophage responses in the context of infection. Thus in CD200 knockout mice there is increased macrophage activity and severe immune-mediated lung damage after influenza infection.^57^

The activation status of NK cells is also controlled by the balance of various inhibitory and activation receptors.^58^, ^59^ For example, the NK cell–activating receptor NKG2D is ligated by molecules, such as MHC class I polypeptide–related sequences A and B or UL16-binding proteins, which are only expressed on stressed airway epithelial cells,^60^, ^61^ resulting in killing of the target cells and ultimately leading to protection from infection. The importance of NK cells and NKG2D in allergic airways responses has been suggested by the findings that mice lacking NKG2D are resistant to induction of allergic inflammation. Although adoptive transfer of wild-type NK cells was able to restore the response, granzyme B–deficient NK cells could not.^62^

One common link between both infectious and noninfectious triggers of type 2 immunity is that many induce some level of physical trauma that breaches the protective barrier of the body. Tissue damage, at least in the absence of strong type 1–promoting ***pathogen-associated molecular pattern*** signaling, appears to be a potent mechanism driving type 2 immunity. This involves rapid release of several epithelium-derived cytokine alarmins, such as IL-1, IL-33, thymic stromal lymphopoietin (TSLP), and IL-25, all of which can drive downstream type 2 immunity.^63^ These cytokines invoke an immune response, involving mast cells, basophils, eosinophils, type 2 innate lymphoid cells (ILC2s), and alternatively activated macrophages, which has evolved to respond to a parasitic infection by generating proinflammatory mediators, toxin-neutralizing enzymes, and helminth-killing toxins, which also have endogenous tissue-damaging properties. A number of studies have identified many environmental agents linked to asthma that have the potential to cause epithelial barrier disruption and tissue injury in the airways, including the house dust mite allergen Der p 1,^64^ fungal allergens,^65^ rhinovirus,^66^ ***cigarette smoke***,^67^, ^68^ and air pollutants.^69^, ^70^

Nonetheless, a key question arising from these observations is the following: Why are the airways of asthmatic subjects more susceptible than normal to these relatively ubiquitous agents? As detailed below, it is likely that the explanation lies in a combination of (1) decreased epithelial barrier defenses reducing the threshold for epithelial damage, (2) dysregulated innate immune or immunoregulatory responses that contribute to ongoing barrier dysfunction, and (3) impaired epithelial barrier repair, leading to failure to resolve inflammatory responses.

## Dysregulation of the epithelial barrier in asthmatic patients

**Fig 3:**
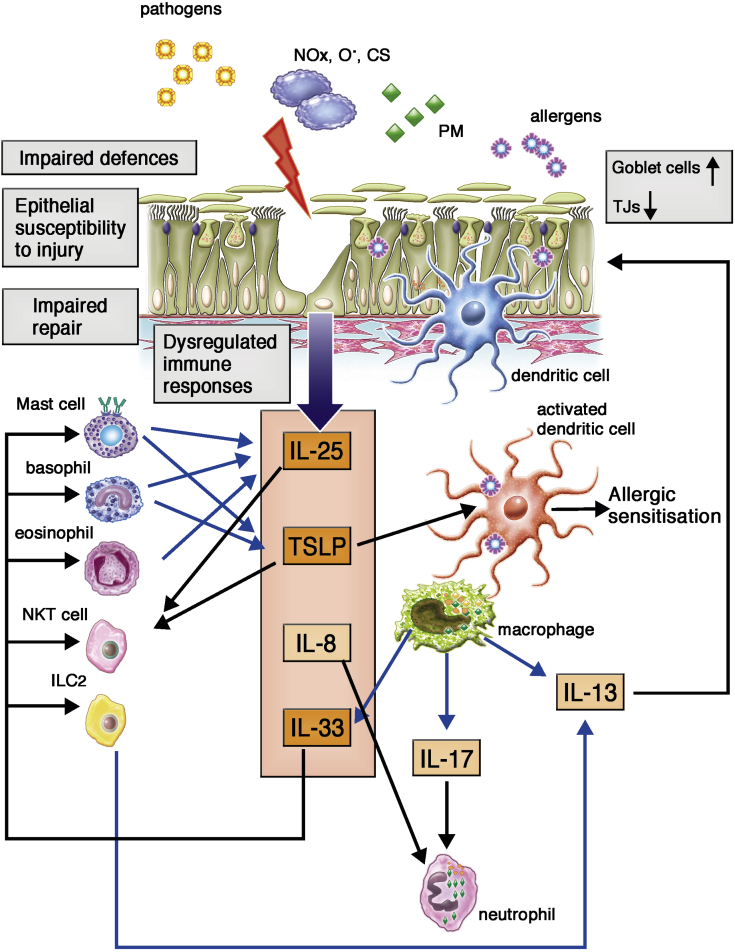
Schematic representation of the epithelial barrier in asthmatic patients highlighting abnormalities in protective and immunoregulatory functions *(gray boxes)*. Persistent airway inflammation most likely arises as a consequence of impaired barrier defenses (altered cytoprotective secretions and reduced cell-cell adhesion), leading to epithelial susceptibility to injury and dysregulated immune responses. In parallel, impaired repair might contribute to maintenance of epithelial activation and chronicity of responses. The relative contribution of each aspect of barrier dysfunction is likely to influence the overall phenotype of the epithelium and might manifest as distinct subgroups of asthma. *CS*, Cigarette smoke; *NO*_*x*_, nitrogen oxides; *O*^*·*^, oxygen radicals; *PM*, particulate matter.

Targeted studies of the bronchial epithelium have demonstrated a range of abnormalities at many levels of barrier function and innate immunity (Fig 3). However, unbiased transcriptomic approaches are now enabling in-depth analysis of epithelial gene expression profiles^8^, ^9^ to provide evidence of molecular mechanisms that might eventually define specific epithelial endotypes of asthma. We will first summarize key abnormalities identified in the epithelial barrier in asthmatic patients and then put these into the context of newer clusters that have been identified and how these relate to genetic susceptibilities.

The mucociliary apparatus is modified in asthmatic patients, as evidenced by an increase in the number of goblet cells with increased mucin gene expression, an increase in MUC5AC protein relative to MUC5B, and a reduction in ciliated cell numbers.^71^, ^72^, ^73^ In addition, decreased ciliary beat frequency, dyskinesia, and ciliary disorientation have been reported in patients with severe asthma.^74^ Together, mucus hypersecretion and ciliary dysfunction in asthmatic patients can result in stimulation of neural receptors that result in cough^75^ and mucous plugging, which, over time, can lead to severe airflow obstruction.

The increase in expression of MUC5AC relative to MUC5B seen in asthmatic patients has been postulated to affect mucus clearance, reduce eosinophil apoptosis,^76^ and/or contribute to abnormal innate immune responses.^57^ Reprogramming of epithelial differentiation toward a hypersecretory phenotype has been linked to increased expression of the epidermal growth factor receptor (EGFR)^72^ and to the activity of T_H_2 cytokines, including IL-13 and IL-9.^77^, ^78^ Consistent with this, patients with T_H_2-high asthma have significantly increased airway mucin gene expression.^79^ T_H_2 cytokines also significantly decrease epithelial expression of the antimicrobial peptide human β-defensin 2 *in vitro*, and mice with allergic airway inflammation have significantly more viable bacteria in their lungs after infection.^80^ In contrast, atopic asthmatic patients with type 2–high asthma have been reported to harbor significantly lower bronchial bacterial burden,^81^ and in patients with severe asthma, no taxa were associated with a T_H_2-related epithelial gene expression signature.^82^ These differences might reflect long-term changes and treatment effects and contrast with the acute responses seen after infection of mice with allergic airways inflammation.^80^

There is considerable evidence for an association between levels of particulate pollutants and asthma exacerbations,^83^, ^84^, ^85^ asthma pathogenesis, and poorer lung function outcomes.^86^, ^87^, ^88^ Exposure to air pollutants can lead to oxidative stress in the airways, and there is compelling evidence that asthmatic airways are deficient in antioxidant defenses.^89^ Furthermore, the antioxidant capacity of the lungs is inversely related to asthma severity.^90^ In addition to lower levels of superoxide dismutase and catalase,^89^ it has been shown that goblet cells express the high-affinity sodium ascorbate cotransporter, which is involved in vitamin C uptake into cells, and that expression of sodium ascorbate cotransporters is inversely related to lung lining fluid vitamin C levels.^91^ There is also considerable evidence that polymorphisms in ***glutathione***-cycling enzymes can result in increased susceptibility to air pollution.^92^, ^93^, ^94^ Glutathione-S-transferase (GST)-pi is predominantly expressed in airway epithelial cells, and expression is decreased in the airways of children with asthma.^95^ In view of the increased susceptibility of the asthmatic bronchial epithelium to oxidant-induced apoptosis *in vitro*^96^ and the observation that increased levels of oxidants can reduce the anti-inflammatory effects of budesonide, an inability to control oxidative stress might not only drive epithelial damage but also confound treatment responses.^97^

***Polyaromatic hydrocarbons*** are a key toxic component of air pollution. Polyaromatic hydrocarbon levels are increased in the plasma of asthmatic children and linked to a number of asthma markers.^98^ The aryl hydrocarbon receptor (AhR), which plays a key role in detoxification of environmental pollutants, also regulates multiciliogenesis.^99^ Importantly, although air exposure triggers AhR targeting of genes important for multiciliogenesis, toxic AhR ligands induce detoxifying cytochromes, with no overlap in target gene induction. These mutually exclusive responses suggest a potential pathophysiologic mechanism whereby AhR ligands in air pollutants disrupt AhR-mediated ciliogenesis to contribute to disruption of barrier defenses in asthmatic patients.^99^

Epithelial fragility^100^ and epithelial shedding^101^ in asthmatic patients have been recognized for many years, but this remains a controversial area.^102^ Nonetheless, through use of specific markers of response to injury, such as increased expression of EGFR, epithelial damage has been confirmed in bronchial biopsy specimens from asthmatic adults^103^ and children.^104^ Many studies have reported disruption of adhesive mechanisms in asthmatic patients, including loss of TJ proteins,^67^, ^105^, ^106^ reduction in AJ proteins,^105^ and reduction in desmosome length.^107^ Membrane expression of caveolin-1, a stabilizer of AJs, is significantly lower in airway epithelia of asthmatic patients, and *in vitro* loss of caveolin-1 causes loss of junctional E-cadherin and β-catenin expression and disrupted epithelial barrier function.^108^ Consistent with reduced adhesion, functional studies comparing epithelial cultures from asthmatic or healthy donors indicate that there is increased permeability and sensitivity to environmental stressors in asthmatic patients^67^ and increased susceptibility to oxidant stress.^96^ Increased barrier permeability might not only promote allergic sensitization but also reduce the threshold for epithelial damage and activation of a type 2 response, which itself might affect barrier function. Thus, in addition to their effects on goblet cell differentiation, T_H_2 cytokines have a disruptive effect on epithelial barrier function^109^ and lead to a distinct profile of epithelial gene expression, both *in vitro* and in T_H_2-high asthmatic patients *in vivo.*^79^

Claudin-18, a lung-specific barrier claudin, has been shown to be expressed in bronchial epithelium, and its levels are reduced in asthmatic patients, being lowest in patients with T_H_2-high asthma.^106^ In the same studies IL-13 downregulated claudin-18 *in vitro*, and targeted knockdown of claudin-18 increased epithelial permeability. Furthermore, claudin-18–null mice had significantly higher serum IgE levels and increased airway responsiveness after intranasal *Aspergillus* species sensitization, suggesting loss of claudin-18 can promote sensitization and airway hyperresponsiveness.^106^

Because mast cells are important sources of IL-13 and are in close proximity to the bronchial epithelium in asthma,^110^ it is noteworthy that IL-33–activated mast cells, as well as ILC2s, are able to drive a predominantly IL-13–regulated pattern of gene expression in normal human bronchial epithelial cells *in vitro.*^111^ Furthermore, ILC2s have been shown to directly impair epithelial barrier integrity through IL-13,^112^ whereas T_H_2 cells cause barrier leakiness through IL-4 and IL-13, an effect that can be prevented by inhibition of histone deacetylases.^113^

Consistent with the evidence of epithelial disruption in asthmatic patients, levels of epithelium-derived cytokine alarmins, such as IL-33, TSLP, and IL-25, are increased in asthmatic patients.^114^, ^115^ IL-33, a member of the IL-1 cytokine family, has gained prominence in type 2 immunity by virtue of the genetic association of both *IL33* and its receptor, *IL1RL1* (ST2), with asthma^10^, ^116^ and by its functional effects on ILC2 cells, T_H_2 cells, mast cells, basophils, and alternatively activated macrophages.^117^ IL-33 is normally localized in the nucleus, where it is a transcriptional regulator^118^ and can act as an extracellular cytokine by binding to its receptor, ST2.^119^ Full-length IL-33 binds ST2 and is biologically active, although activity can be increased after cleavage by inflammatory proteases,^120^ whereas caspase cleavage leads to inactivation.^121^ IL-33 can be released by nonprogrammed cell death, or it can be actively secreted through vesicular transport from the Golgi complex.^122^ Stimulation of bronchial epithelial cells with allergen or ATP results in active release of IL-33, which depends on the NADPH oxidase dual oxidase 1 (DUOX1)–mediated activation of Src and EGFR signaling through cysteine oxidation.^123^ Nasal epithelial cells from asthmatic patients display enhanced DUOX1 expression, as well as allergen-induced IL-33 secretion, compared with healthy control subjects, suggesting that increased expression and activation of DUOX1 might be an important feature of enhanced IL-33 secretion in asthmatic patients.^123^ In addition to full-length IL-33, alternative splicing of the IL-33 transcript can result in deletion of exons 3 and 4 (Δ exon 3,4) to confer cytoplasmic localization and facilitate extracellular secretion without cell death, while retaining signaling capacity. Analyses of epithelial brush RNA suggest that Δ exon 3,4 is strongly associated with airway type 2 inflammation, whereas full-length IL-33 is not.^124^ These results suggest that therapeutic IL-33 inhibitors will need to block all biologically active isoforms.

TSLP is an IL-7–like cytokine that can trigger DC-mediated T_H_2 inflammatory responses^125^ and T_H_2 cytokine production by mast cells.^126^ A variety of stimuli, including double-stranded RNA and allergens, stimulate TSLP expression in bronchial epithelial cells, and this is enhanced by inflammatory cytokines.^127^ Challenge of cultured epithelial cells from asthmatic donors with double-stranded RNA results in a skewed response favoring more TSLP and less type 1 interferon compared with healthy cells.^128^ Allergen-specific T cells also enhance TSLP production by epithelial cells from asthmatic donors, suggesting that T cell–airway epithelium interactions can lead to maintenance and amplification of allergic inflammation.^129^ In a double-blind, placebo-controlled study, treatment with a human mAb to TSLP resolved airway inflammation and attenuated allergen-induced bronchoconstriction, findings consistent with TSLP as a therapeutic target in patients with allergic asthma.^130^ However, in addition to its effects on immune cells, it is noteworthy that TSLP drives an IL-13–dependent increase in bronchial epithelial cell proliferation^131^ and increases TJ expression to enhance nasal epithelial barrier function, suggesting a role for TSLP in restoration of epithelial barrier integrity.^132^ In contrast, TSLP has been reported to disrupt TJs in 16HBE bronchial epithelial cells.^133^ Furthermore, a short and constitutively expressed form of TSLP has been detected in the skin and gut; this variant cannot activate signal transducer and activator of transcription (STAT) 5 but has potent antimicrobial activity.^134^ Recent studies suggest that the short and constitutively expressed form of TSLP can protect against bronchial epithelial barrier disruption *in vitro* and house dust mite– or toluene diisocyanate–induced airway inflammation *in vivo*.^133^, ^135^ Consequently, optimal therapeutic antibody targeting might need to be directed specifically to the long form of TSLP.

IL-25 belongs to the IL-17 cytokine family and is secreted by T_H_2 cells, mast cells, basophils, and eosinophils, as well as epithelial cells.^136^ It can drive airway remodeling in allergic models of airway inflammation,^137^ and, in combination with IL-33, can promote the development of ILC2s, which appear critical in early initiation of the T_H_2 response.^138^ Expression of IL-25 has been reported to be increased in epithelial cells from patients with asthma and can be induced further by rhinovirus infections.^139^ Others have found increased systemic levels of IL-25 in subgroups of patients with T_H_2-high asthma.^140^ Furthermore, the IL-25 receptor (IL-17RB) is upregulated on myeloid and plasmacytoid DCs in blood and sputum 24 hours after allergen challenge.^141^ IL-25 upregulated TLR9 expression by plasmacytoid DCs and orchestrated the responses to TLR9 ligation, suggesting that IL-25 can act as a link between the adaptive and innate immune responses.^141^

Viral respiratory tract infections, especially rhinovirus infection, are the main triggers of asthma exacerbations.^142^, ^143^ Several,^144^, ^145^, ^146^ but not all,^147^, ^148^ studies have shown that bronchial epithelial cells from asthmatic donors respond abnormally to rhinovirus infection involving an insufficiency of IFN-β and IFN-λ. This has been linked to increased TGF-β2 production by epithelial cells from asthmatic patients^149^ and suppression of cytokine signaling expression^150^; however, it is also of interest that rhinovirus-induced EGFR activation can suppress IFN-λ production and increase viral infection.^151^ The importance of decreased antiviral immunity in asthmatic patients has been tested in a clinical trial with inhaled IFN-β: the drug was found to improve asthma control and reduce exacerbations in patients with difficult-to-treat asthma.^152^

It is well known that mechanical forces are critical to lung development and that abnormal mechanical stresses can lead to pathologic lung injury.^153^ In asthmatic patients constriction of bronchial smooth muscle during an acute asthma attack causes the airway wall to buckle, resulting in folding and compression of the bronchial epithelium.^153^ *In vitro* studies have shown that airway epithelial cells respond rapidly and robustly to compressive stress with changes in goblet cell numbers and production of profibrogenic growth factors.^154^, ^155^ The relevance of these findings has been demonstrated *in vivo*, where induction of bronchoconstriction with methacholine caused airway remodeling involving goblet cell metaplasia and subepithelial fibrosis without evidence of inflammation.^156^ Although these changes might simply be due to the hyperresponsive properties of bronchial smooth muscle in asthmatic patients, there is evidence that bronchial epithelial cells from asthmatic donors respond abnormally to compression with increased release of TGF-β and GM-CSF,^157^ suggesting that bronchoconstriction can skew epithelial innate immune responses in asthmatic patients. Because the asthma susceptibility gene a disintegrin and metalloprotease 33 *(ADAM33)* has been linked to BHR^158^ and has been shown to cause bronchial smooth muscle contraction,^159^ there is the potential for multifactorial indirect genetic effects on epithelial barrier function.

Increased expression of the EGFR in bronchial biopsy specimens from asthmatic adults^103^ and children^104^ is consistent with an ongoing response to injury, and this is highly correlated with epithelial IL-8 expression.^160^ However, expression of the cyclin-dependent kinase inhibitor p21^waf^ might be indicative of impaired proliferation or ongoing epithelial stress in asthmatic patients.^104^, ^161^ During epithelial repair, neighboring epithelial cells become migratory in response to growth factors, such as TGF-β or epidermal growth factor. This repair phenotype is characterized by downregulation of TJs and increased expression of matrix metalloproteases and extracellular matrix components, as observed in asthmatic patients. Studies with cultures of epithelial cells from asthmatic children suggest that the airway epithelium displays a dysregulated repair response, taking longer to repair mechanically induced wounds^162^ and undergoing a more extensive epithelial-mesenchymal transition in response to TGF-β than cultures from nonasthmatic donors.^163^ Recently, it has been reported that IL-22 can promote a repair phenotype in the presence of TGF-β1, causing a marked reduction in E-cadherin but only in cells obtained from donors with severe asthma.^164^

## Epithelial clusters and asthma heterogeneity

**Fig 4:**
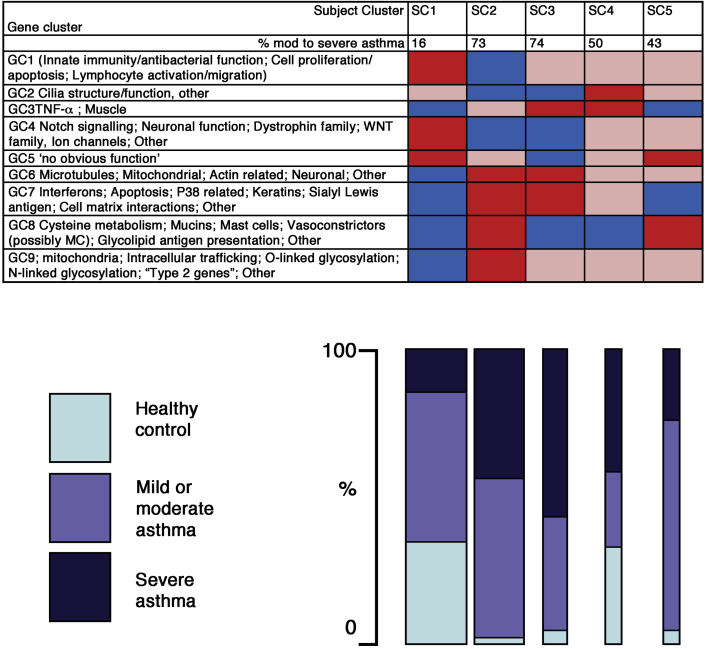
Pictorial representation of the SCs and GCs found in a transcriptomic analysis of epithelial brushings from 155 donors. Red indicates high, pink indicates medium, and blue indicates low expression of genes within the cluster. The *bar chart* indicates the percentage of healthy control subjects and patients with mild, moderate, or severe asthma in each SC, and the width of the bar is proportional to the number of subjects in the cluster. Findings are summarized from Modena et al.^8^

Use of large-scale transcriptomic approaches in large cohorts of well-characterized asthmatic and healthy control volunteers has enabled unbiased in-depth analysis of gene expression profiles in epithelial brushings and allowed clustering into distinct phenotypes. Analysis of transcriptomic data from 155 donors in combination with exhaled nitric oxide has identified 5 molecularly defined and clinically distinct subject clusters (SCs) with distinct expression of gene clusters (GCs),^8^ as summarized in Fig 4. The majority (73%) of all healthy control subjects were located in SC1, which was distinguished by high expression of GCs involved in processes including “innate immunity/antibacterial function” and “Notch signaling” and low expression of GCs, including “interferons/stress” and “type 2 immunity.” In contrast, the largest group of patients with severe asthma (SC2) showed a diametrically opposite pattern with low expression of both “innate immunity/antibacterial function” and “Notch signaling” GCs and high expression of “interferons/stress” and “type 2 immunity” GCs. In addition, “cilia structure and function” was low in SC2 with severe asthma. It is interesting to note an apparent paradox that gene signatures for both cilia-related genes and Notch signaling are reduced in SC2. Because Notch signaling inhibits ciliated cell differentiation *in vitro* by repressing multicilin and forkhead box J1,^165^ low Notch levels might suggest increased ciliogenesis, but this was not the case. However, it has been shown that IL-13 inhibits ciliated cell differentiation independent of Notch signaling,^166^ suggesting 2 distinct signaling pathways can affect ciliated cell differentiation, which might be of relevance in the different SCs of severe asthma. The other SCs showed some overlap with SC2, but each exhibited distinct profiles illustrating the heterogeneity of the epithelial gene signature across the spectrum of asthma severity. Further analysis of the same data using weighted gene coexpression network analysis (WGCNA) highlighted that genes in modules linked to epithelial growth and repair and neuronal function were markedly decreased in patients with severe asthma.^9^ Of particular note, low expression of epithelial growth and repair and neuronal function genes was more strongly associated with severe asthma than type 2 inflammation, suggesting that epithelial integrity and related processes are of primary importance to the development of asthma and severe asthma.

Assuming that these phenotypes are stable rather than fluctuations because of disease activity, these data illustrate the complexity of the epithelial phenotype. Reinforcement of these findings with longitudinal studies should provide a basis for hypothesis-driven research that allows precise definition of epithelial endotypes in asthmatic patients. Nonetheless, based on the evidence to date, further consideration of strategies that promote epithelial repair and restore epithelial homeostasis might provide novel therapeutic approaches for the treatment of asthma.^24^ For example, the protective effects of growth factors, such as epidermal growth factor, have been recognized for many years (reviewed by Swindle et al^24^). However, novel strategies include potential use of the macrolide antibiotic azithromycin, which has been shown to decrease ionic permeability of human airway epithelia by changing the processing of TJ proteins,^167^ or histone deacetylase inhibition with JNJ-26481585, which has been shown to ameliorate the effects of T_H_2 cells on barrier function.^113^

## From asthma genes to function

**Fig 5:**
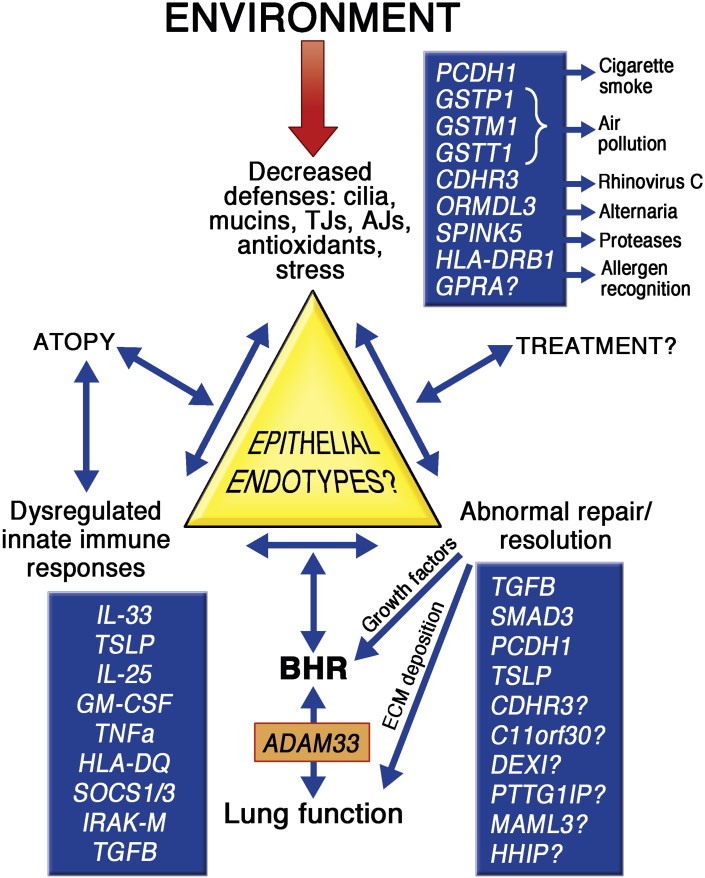
Potential mechanisms of asthma defined by epithelial barrier dysfunction. Identification of potential links with asthma susceptibility genes and their interaction with environmental stimuli are shown.

Genome-wide association studies (GWASs) of asthma have identified novel risk alleles and loci, with many of the asthma susceptibility genes being expressed in the airway epithelium.^168^ Among susceptibility factors for asthma, the genes *IL1RL1*/*IL18R1*, *IL33*, and *TSLP* have emerged as some of the most important associated with development of the disease,^10^ linking epithelium-derived cytokines to type 2 inflammation. Furthermore, a number of genes associated with epithelial homeostasis, differentiation, or barrier immunity have been identified, including protocadherin 1 *(PCDH1)*,^169^ cadherin-related family member 3 *(CDHR3)*,^170^ *HLA-DQ*,^10^ *SPINK5*,^171^ *GPRA*,^172^ and orosomucoid-like 3 *(ORMDL3)/GSDMB*^10^ at the 17q12-21 locus. However, it should be noted that asthma-associated alleles have small effect sizes and account for little of the prevalence of asthma, and it is likely that a significant portion of the genetic risk for asthma and its exacerbations results from genotype-specific responses to environmental exposures, including allergens, pollution, and viral infections, especially at particular stages of life.^173^, ^174^, ^175^, ^176^, ^177^ Here we have attempted to place some of the asthma susceptibility genes in the context of epithelial barrier dysregulation, with a view to highlighting potential epithelial endotypes of disease linked to reduced barrier defenses, dysregulated immune responses, and/or abnormal repair responses (Fig 5).

Epidemiologic and genetic evidence have implicated epithelial susceptibility to environmental insults in asthma pathogenesis. However, clear functional relationships are not always easy to identify, perhaps reflecting the need for assessment in the context of an appropriate environmental trigger. For example, although 2 common deletion polymorphisms of the GST genes *GSTM1* and *GSTT1* and the *GSTP1* Ile105Val polymorphism have been associated with asthma in children and adults, a meta-analysis has revealed extreme between-study heterogeneity,^178^ suggesting more focused study in the context of environmental oxidative exposures would be more informative.

Genes, such as the cadherin family members *CDHR3*^170^ and *PCDH1*,^169^ appear to play roles in adhesion. Several single nucleotide polymorphisms (SNPs) in *PCDH1* have been linked to asthma and BHR. These include Ala750Ala and IVS3_116, which are localized in the 3′ untranslated region of exon 3 and might affect mRNA stability or splicing, whereas Ala514Thr is localized in the fifth cadherin repeat of the extracellular domain and can affect cell-cell adhesion^169^; however, the functional consequences of this mutation have not been explored. Protocadherin 1 (PCDH1) colocalizes with E-cadherin in airway epithelial cells, and it has been implicated in the barrier-enhancing properties of glucocorticoids^179^ and suppression of TGF-β signaling.^180^ Because gene–passive smoking interactions have been found to be relevant for the association of *PCDH1* with asthma,^169^, ^181^ the contribution of *PCDH1* gene variants to asthma might only become evident in the context of smoke exposure.^182^ *CDHR3* was originally identified as an asthma susceptibility gene linked to childhood exacerbation.^170^ The asthma-associated SNP (rs6967330) causes a nonsynonymous mutation (G>A; C529Y) in the fifth cadherin repeat of CDHR3, which affects cellular localization.^170^ Subsequent studies showed that CDHR3 is a receptor for rhinovirus C, suggesting that the increased localization of Y529 CDHR3 on the bronchial epithelial cell surface increases susceptibility for rhinovirus C infection and replication.^183^ However, the normal cellular function of CDHR3 is still unknown.

*ORMDL3* has been shown to be associated with early-onset asthma susceptibility in multiple independent genome-wide and candidate-gene association studies.^173^ It is regulated by STAT6 and can be induced by IL-13 or IL-4,^184^ and SNPs in *ORMDL3* correlate with changes in T_H_2 cytokine levels.^185^ ORMDL3 is found in the endoplasmic reticulum and is involved in maintaining sphingolipid homeostasis and in the unfolded protein response,^186^ but *in vitro* studies involving underexpression or overexpression of ORMDL3 did not show a significant role in modulating innate immune responses and the unfolded protein response.^187^ However, in mice overexpression of ORMDL3 decreases serum sphingolipid levels and increases inflammatory markers, airway remodeling, and BHR in response to allergic stimuli.^188^ Furthermore, pulmonary epithelial expression of ORMDL3 is sufficient for induction of *Alternaria* species–induced allergic airways disease.^189^

As already described, polymorphisms in genes, including *IL33*, *IL1RL1*, and *TSLP*, have been linked to epithelial activation/damage and type 2 immunity, although detailed studies are still revealing new levels of complexity involving alternative splicing.^124^ In the case of TSLP, multiple SNPs are correlated with the expression levels of TSLP, and some alleles are protective.^190^ Of note, in subjects with 1 or more *SPINK5* risk alleles, the absence of the TSLP protective minor alleles has been associated with a significant increase in asthma.^191^ Thus, in addition to gene-environment effects, ***epistasis*** adds another level of complexity to asthma pathogenesis. Other immune regulators might be relevant to exacerbation-prone asthma: these include suppressor of cytokine signaling 1 *(SOCS1)*^192^ and IL-1 receptor–associated kinase M *(IRAK-M)*,^193^ both of which suppress IFN-β signaling and antiviral responses.^150^, ^194^

The focus on epithelial repair genes in asthmatic patients has been limited to date, but promoter variants in *TGFB1* and *TGFB2*, which increase TGF-β expression, are associated with asthma^195^, ^196^ and airflow obstruction.^197^ It is also interesting to note that genes, such as hedgehog interacting protein *(HHIP)* and patched homolog 1 *(PTCH1)*, which might play a role in epithelial repair have been identified through genetic association with reduced lung function,^198^ suggesting that impaired repair might drive extracellular matrix deposition and tissue remodeling.

Most of the asthma-associated SNPs identified by using GWASs are not coding-change variants. Therefore expression quantitative trait loci (eQTLs) analysis has been adopted to identify functional SNPs regulating expression levels of disease-associated genes in a cell type–specific fashion. Applying this analysis to bronchial epithelial cells has revealed SNPs in *TSLP*, *GSDMB*, *IL33*, *HLA-DQB1*, *C11orf30*, *DEXI*, *CDHR3*, and *ZBTB10* that affect asthma risk by allowing *cis*-regulation of its gene expression in an epithelial specific manner.^190^ In the case of *IL33*, all asthma-associated SNPs in this region of the genome are located in the 5′ or first intron of *IL33*, and eQTL analysis has revealed that SNPs in the promoter region of *IL33* are correlated with IL-33 expression in bronchial epithelial cells. The same study identified an eQTL SNP for *CDHR3* (rs17152490) in bronchial epithelial cells, which is in linkage disequilibrium with the GWAS SNP (rs6967330, G>A; C529Y), suggesting *cis*-regulation of *CDHR3* expression can also contribute to the asthma risk. SNPs in pituitary tumor–transforming 1 interacting protein *(PTTG1IP)* and mastermind-like 3 *(MAML3)* have been reported to be associated with BHR severity in adult asthma,^199^ and eQTL analyses indicate higher tissue expression with less severe BHR. These gene products might be particularly relevant to epithelial repair because PTTG1lP is coexpressed with vimentin and E-cadherin 1, whereas MAML3 is coexpressed with MAML2, both of which are involved in Notch signaling, a repair pathway that was deficient in the transcriptomic studies of severe asthma.

## Concluding comments

Taken together, the evidence for epithelial dysregulation in asthmatic patients is compelling. Genomic studies have revealed the extent of epithelial heterogeneity in asthmatic patients and have provided considerable insight into expression profiles, pathways, and processes that can drive epithelial dysfunction. Further understanding of asthma endotypes will come from integration of findings from these large data sets with the function and regulation of asthma genes and how these are modified by interaction with environmental factors, including the airway microbiome. However, the stability of the asthma phenotypes identified in molecular studies still needs to be addressed in longitudinal studies. In addition, the appreciation that changes in gene expression are also evident in epithelial cells harvested from peripheral airways of patients with severe asthma raises new questions about gene dysregulation in the smaller airways, which comprise the majority of the airway surface area, and the need for better-targeted therapies for the peripheral airways.^200^ Furthermore, there is a lack of critical information about epithelial heterogeneity and its role in childhood asthma. Crucially, we still lack detailed information about the functions of many asthma genes and how genetic polymorphism of these genes drives asthma susceptibility. The high costs of transgenic and gene-deletion mouse models has restricted progress in this area. Thus it would be timely to investigate the potential of nonmammalian models, such as *Drosophila* species or zebrafish, as tools to investigate gene function because the genetic tractability and low cost of rearing these organisms are major advantages.^201^, ^202^ Better understanding of epithelial dysfunction and its interrelationship with airways inflammation and structural remodeling should help to define specific epithelial endotypes in asthmatic patients. Through development and use of therapeutic approaches that restore epithelial barrier homeostasis, it might be possible to prevent or modify the disease course by intervening close to the origin of the disease.
